# Cytokine levels in breast cancer are highly dependent on cytomegalovirus (CMV) status

**DOI:** 10.1007/s10549-024-07459-8

**Published:** 2024-08-22

**Authors:** Juliet V. Spencer, Jianfang Liu, Brenda Deyarmin, Hai Hu, Craig D. Shriver, Stella Somiari

**Affiliations:** 1 Department of Biology, Texas Woman’s University, Denton, TX USA; 2 Chan Soon-Shiong Institute of Molecular Medicine at Windber, Windber, PA USA; 3 Murtha Cancer Center, Uniformed Services University of the Health Sciences, Bethesda, MD USA

**Keywords:** Breast cancer, CMV, Cytomegalovirus, Cytokine, In situ carcinoma, Invasive carcinoma

## Abstract

**Purpose:**

Breast cancer accounts for 30% of all female cancers in the US. Cytomegalovirus (CMV), a herpesvirus that establishes lifelong infection, may play a role in breast cancer. CMV is not oncogenic, yet viral DNA and proteins have been detected in breast tumors, indicating possible contribution to tumor development. CMV encodes cmvIL-10, a homolog of human cellular IL-10 (cIL-10) with potent immunosuppressive activities. We investigated the relationship between CMV infection, cytokines, and breast cancer.

**Methods:**

We evaluated CMV serostatus and cytokine levels in plasma of women with benign breast disease (*n* = 38), in situ carcinoma (*n* = 41), invasive carcinoma, no lymph node involvement (Inv/LN−; *n* = 41), and invasive with lymph node involvement (Inv/LN+; *n* = 37).

**Results:**

Fifty percent of the patient samples (*n* = 79) were CMV seropositive. There was no correlation between CMV status and diagnosis (*p* = 0.75). For CMV+ patients, there was a trend toward higher CMV IgG levels in invasive disease (*p* = 0.172). CmvIL-10 levels were higher in CMV+ in situ patients compared to the Inv/LN− and Inv/LN+ groups (*p* = 0.020). Similarly, cIL-10 levels were higher in CMV+ in situ patients compared to the Inv/LN− and Inv/LN+ groups (*p* = 0.043). The results were quite different in CMV− patients where cIL-10 levels were highest in Inv/LN− compared to benign, in situ, or Inv/LN+ (*p* = 0.019). African American patients were significantly associated with CMV+ status (*p* = 0.001) and had lower cmvIL-10 levels than Caucasian patients (*p* = 0.046).

**Conclusion:**

No association was observed between CMV IgG and diagnosis, but CMV infection influences cytokine production and contributes to altered cytokine profiles in breast cancer.

## Introduction

Breast cancer is the second leading cause of cancer deaths for women in the United States. Although women with localized tumors have a 5-year survival rate near 100%, women with invasive tumors that spread to distant sites have a 5-year survival rate of only 30% [1, 2]. Identification of factors that promote in situ tumors to become invasive is necessary to improve treatment options and increase overall survival in breast cancer patients.

One factor linked to invasive breast cancer is human cytomegalovirus (CMV) [3–5]. CMV is a herpesvirus that establishes lifelong latent infection in 70% to 90% of the general population [6]. CMV periodically reactivates but typically causes clinical disease only in immunocompromised people. However, over a lifetime, CMV may reduce immune function [7–9], contribute to chronic conditions [10–12], and promote tumor progression [13–16].

CMV has been associated with tumor progression and metastatic spread [17]. For example, CMV antibody levels are higher in women with invasive breast cancer than in healthy women [18, 19]. Also, CMV DNA and proteins have been found in tumor biopsy samples and are associated with more invasive tumors [5, 20], higher tumor grade [21], and poor overall survival [22, 23]. Microbiome analysis of women with breast cancer revealed high levels of CMV [24]. CMV broadly influences host immune responses and may impact the ability of cancer cells to evade immune detection [25]. Of note, CMV encodes cmvIL-10, a viral cytokine and ortholog of human cellular interleukin-10 (cIL-10) with potent immune suppressive properties [26–29]. Immune suppression in the tumor microenvironment is a key factor in progression from localized to invasive tumors [30].

While cmvIL-10 is best known for immune suppression, this viral cytokine can also stimulate growth of tumor cells [31, 32]. CmvIL-10 that was produced by patient-derived glioma stem cells in culture was shown to enhance cell migration and invasion, thereby influencing the progression of malignant glioma [32]. Also, breast cancer cells exposed to cmvIL-10 in vitro exhibited enhanced cell proliferation, migration, and Matrigel invasion [33–35]. CmvIL-10 has the potential to impact any cell expressing the cIL-10 receptor, including tumor cells and the immune cells responding to the tumor.

To learn whether high levels of cmvIL-10 were associated with invasive breast cancer, we examined CMV serostatus and cytokine levels in a population of women with varying stages of breast disease. Previously collected plasma samples from patients with benign breast disease, in situ carcinoma, invasive carcinoma with no lymph node involvement (Inv/LN−), and invasive carcinoma with lymph node involvement (Inv/LN+) were evaluated by ELISA for CMV IgG, cmvIL-10, and human cIL-10, IL-6, and TNFα. We hypothesized that cmvIL-10 and cIL-10 levels would be highest in samples from patients with more invasive breast disease, indicating an immune suppressive environment that enabled tumor cells to escape ductal walls and invade surrounding tissue.

## Methods

### Patient samples

Samples for the study were from the CBCP biobank, a large tissue and blood repository of samples from women undergoing treatment for various types of breast disease. Plasma specimens were selected from retrospective collections of the CBCP. Based on disease status, they were grouped as benign (*n* = 38), in situ carcinoma (*n* = 41), invasive carcinoma with no lymph node involvement (*n* = 41) and invasive carcinoma with lymph node involvement (*n* = 37). Plasma was collected from most patients at the time of diagnosis (*n* = 105). In some patients with invasive carcinoma (Inv/LN− or Inv/LN+), plasma was collected following chemotherapy treatment (*n* = 52).

### Enzyme linked immunosorbent assay (ELISA)

Plasma specimens were analyzed for CMV IgG via commercial assay (Trinity Biotech, Jamestown, NY) according to the manufacturer’s instructions. Briefly, the Immune Status Ratio (ISR) was determined using a ratio of optical density readings from the sample and a calibrator. Cytokine levels were analyzed using Human cIL-10, IL-6, and TNFα Duo Set ELISA kits according to the manufacturer’s instructions (R&D Systems, Minneapolis, MN). Measurement of cmvIL-10 was performed via ELISA using goat polyclonal antiserum as described previously [36]. For all cytokine ELISAs, protein concentrations (pg/ml) were determined by interpolation from a standard curve. Cytokine values measured in pg/ml were natural log transformed for statistical analyses.

### Statistical analysis

Cytokine values were log transformed and analyzed using R statistical software. Statistical tests were applied to these data. The Wilcoxon rank sum test was used for testing the difference of numeric variables across two groups. The Chi-squared test and Fisher’s exact test were applied to evaluate the association of two categorical variables. One-way Analysis of Variance (ANOVA) and Kruskal–Wallis rank sum test (non-parametric test for ANOVA) were used to test numeric differences between multiple groups. When the normality assumption of ANOVA was not met, the Kruskal–Wallis rank sum test was used. Linear regression was performed to test the difference between disease diagnoses or evaluate the effect of sample collection time (at diagnosis vs after treatment).

## Results

### Study population

A total of 157 patient plasma samples were obtained from the CBCP biobank. The characteristics of the study population are shown in Table 1. The mean age of all patients included in this study at time of diagnosis was 51.0 years. The majority of patient samples were collected at time of diagnosis. For some patients with a diagnosis of invasive carcinoma (Inv/LN− or Inv/LN+), plasma samples were collected post treatment (Table 2).

**Table 1:** Characteristics of the study population

	Total	CMV+	CMV−	*p-*value
	(*n* = 157)	(*n* = 79)	(*n* = 78)	
Age at diagnosis Mean ± SD (years)	51.0 ± 10.3	52.2 ± 10.6	49.9 ± 9.8	0.12^a^
Race	No. (%)	No. (%)	No. (%)	< 0.001^b^
African American	22 (14%)	20 (25%)	2 (3%)	
Caucasian	127 (81%)	52 (66%)	75 (96%)	
Other	8 (5%)	7 (9%)	1 (1%)	
Tumor				0.75^c^
Benign	38 (24%)	22 (28%)	16 (21%)	
In situ	41 (26%)	20 (25%)	21 (27%)	
Invasive (LN−)	41 (26%)	19 (24%)	22 (28%)	
Invasive (LN+)	37 (24%)	18 (23%)	19 (24%)	
Cytokines Mean ± SD (pg/ml)				
cmvIL-10	–	66.5 ± 198.1	–	
cIL-10	44.0 ± 152.5	47.0 ± 134.5	41.0 ± 169.7	0.41^a^
TNFα	17.3 ± 52.0	19.4 ± 54.1	15.2 ± 50.2	0.76^a^
IL-6	7.3 ± 26.4	7.0 ± 17.6	7.5 ± 33.2	0.71^a^

*No.* number of patients, % percent of total patients in the group

^a^Wilcoxon rank sum test, ^b^Fisher’s exact test, ^c^Chi-squared test

**Table 2:** Evaluation of invasive (Inv/LN− and Inv/LN+) samples by recurrence and collection time

Analysis of all invasive cases	CMV+ (*n* = 37)		CMV− (*n* = 41)	
Recurrence/distant metastases/ contralateral breast involvement?	Yes	No	Yes	No
Plasma collected at Diagnosis	0	17	1	8
Plasma collected post-Treatment	6	14	8	24

Cytokine analysis	cmvIL-10		cIL-10		TNFα		IL-6	
At diagnosis vs. post-treatment	Univ^a^	Adjust^b^	Univ	Adjust	Univ	Adjust	Univ	Adjust
CMV+, non-recurring (17 vs 14)	0.459	0.859	0.423	0.576	0.622	0.711	0.487	0.942
CMV−, non-recurring (8 vs 24)	0.959	0.700	0.625	0.942	0.277	0.461	0.53	0.751
All CMV+ (17 vs 20)	0.734	0.949	0.423	0.569	0.587	0.862	0.315	0.578
All CMV− (9 vs 32)	0.842	0.857	0.494	0.913	0.122	0.236	0.369	0.489

^a^Univariable indicates *p* value for comparison between groups based on plasma collection time, for each cytokine

^b^Adjustment indicates *p* value for comparison of plasma collection time, adjusted by Inv/LN− and INV/LN+

To learn which patients harbored human CMV, plasma samples were tested for immunoglobulin G (IgG) specific for CMV. Patients were designated as CMV seropositive (CMV+) or seronegative (CMV−) based on the presence of CMV IgG. Fifty percent of the samples (*n* = 79) were CMV+ (Table 1). There was no significant difference in age at time of diagnosis between the CMV+ (52.2 ± 10.6) and CMV− (49.9 ± 9.8) groups. Likewise, there was no association between disease diagnosis and CMV status (*p* = 0.75). Race was significantly associated with CMV serostatus, and African American patients were predominantly CMV+ (20/22 participants) (*p* < 0.001).

### CMV IgG levels

**Fig. 1:**
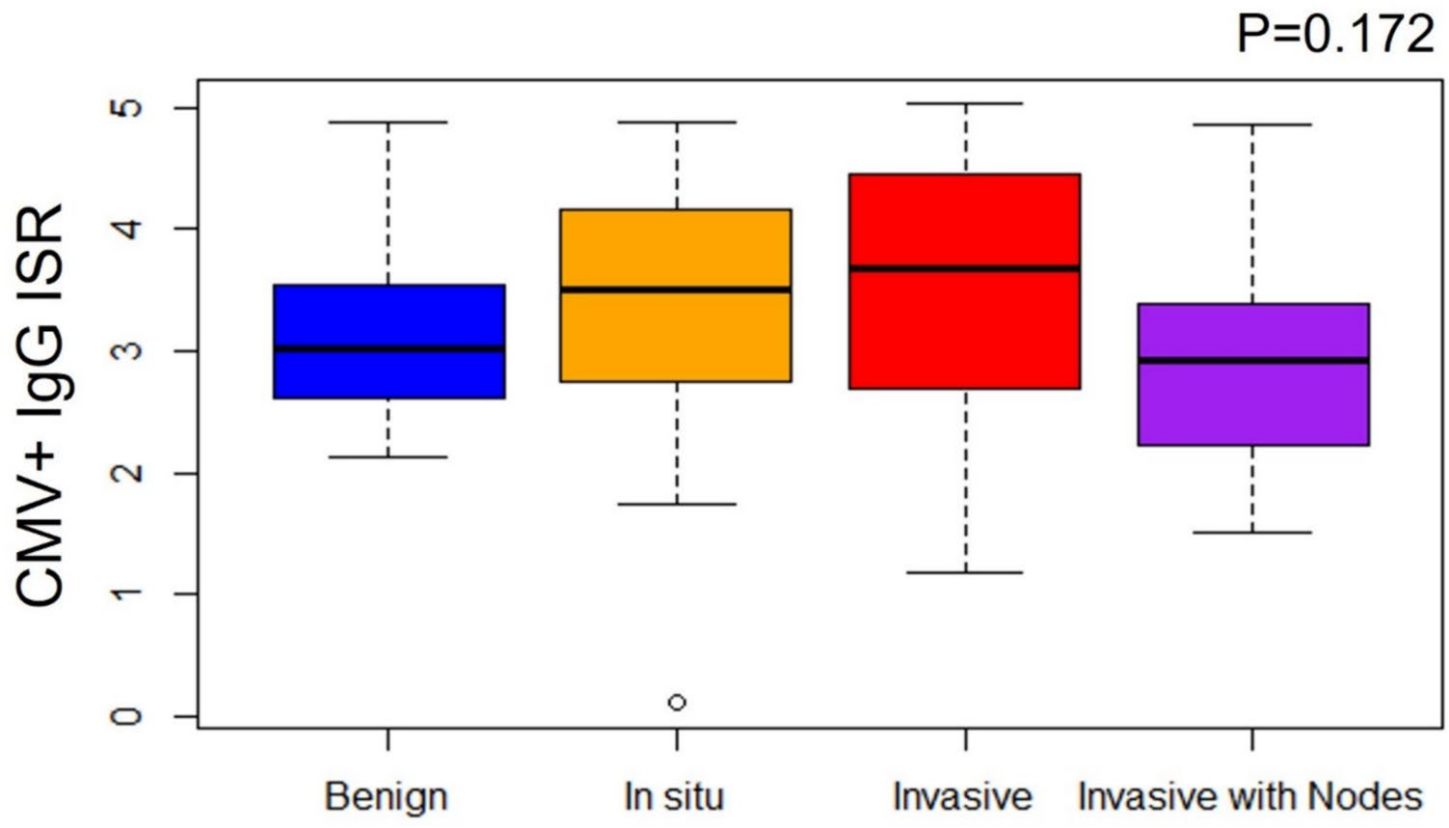
Plasma CMV + IgG levels by breast disease diagnosis. Values represented as immune status ratio (ISR), > 1.1 = seropositive. Only CMV+ patients (*n* = 79) are represented. Statistical analysis by Kruskal–Wallis rank sum test

We next examined whether CMV IgG levels were associated with disease severity by evaluating the Immune Status Ratio (ISR). An ISR value over 1.1 is considered seropositive, but many patients have ISR values well above this level. Higher ISR values could indicate elevated levels of circulating IgG specific to CMV due to recent infection or virus reactivation. No significant differences in CMV + IgG levels were detected between the disease groups (Fig. 1, *p* = 0.172). However, a trend toward higher CMV + IgG levels in the Inv/LN− group was observed.

### Cytokine levels

**Fig. 2:**
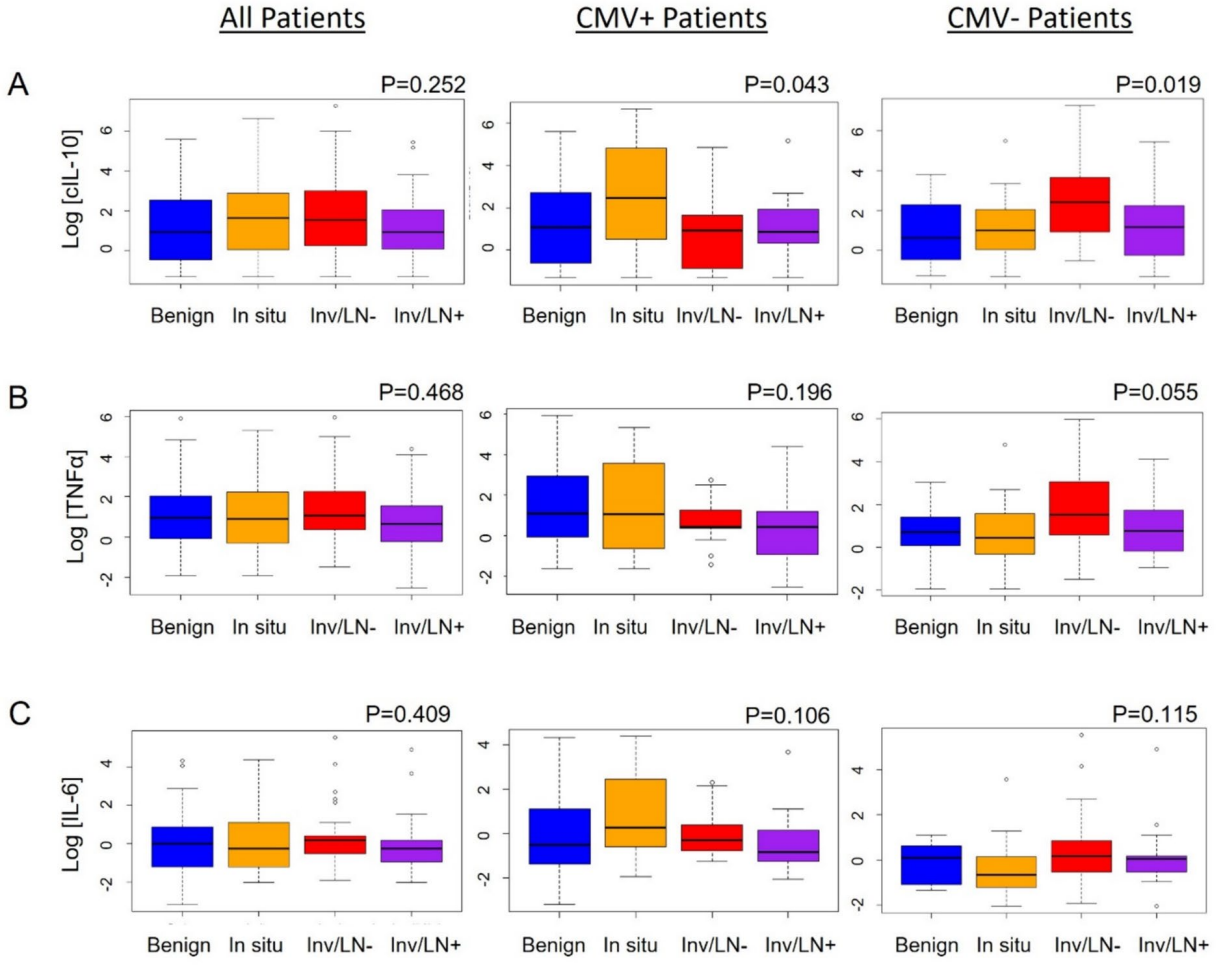
Log cytokine levels in patients with breast disease. **A** Human cIL-10 levels for all patients in the study (*n* = 157), CMV+ patients only (*n* = 79), or CMV− patients (*n* = 78). **B** TNFα levels for all patients, CMV+, or CMV− patients. **C** IL-6 levels for all patients, CMV+, or CMV− patients. All statistical analyses were performed by one-way ANOVA (**A**, **B**) or Kruskal–Wallis one-way ANOVA (**C**)

To learn whether cytokine levels were associated with disease severity, we measured three human cytokines in patient plasma samples: cIL-10, TNFα, and IL-6. There were no significant differences in human cIL-10 levels between disease groups in the overall study population (*p* = 0.252) (Fig. 2A). However, there were significant differences in cIL-10 levels among CMV+ patients (*p* = 0.043). CMV+ patients with a diagnosis of in situ carcinoma had higher cIL-10 levels than those with Inv/LN−, Inv/LN+, or Inv/LN− and LN + combined (*p* = 0.008, 0.031, and 0.005, respectively) (Table 3). Surprisingly, the results were quite different in the CMV− population. A difference was detected across the patient groups (*p* = 0.019); specifically, cIL-10 levels were significantly higher in patients with Inv/LN− compared to those with benign disease, in situ, or Inv/LN+ (*p* = 0.010, 0.011, and 0.012, respectively) (Table 3). In summary, the highest cIL-10 levels were observed in the in situ group for CMV+ patients, whereas cIL-10 levels were highest in the Inv/LN− group for CMV− patients (Fig. 2A).

**Table 3:** Summary of comparisons between disease groups

Log trans-formed value	Population	One-way ANOVA (p)	Linear regression (p)							Comment
		Across all types of breast disease	In Situ vs Inv/LN−	In Situ vs Inv/LN+	In Situ vs all Invasive	In Situ vs Benign	Inv/LN− vs Benign	Inv/LN+ vs Benign	Inv/LN− vs Inv/LN+	
CMV IgG	All	0.534^a^								
	CMV+	**0.172**^a^	0.339	0.865	0.637	0.517	0.732	0.422	0.272	Trend toward higher CMV IgG in patients with Inv/LN− breast disease
	CMV−	0.490^a^	0.980	0.518	0.713	0.368	0.346	**0.136**	0.524	
cIL-10	All	0.252								
	CMV+	*0.043*	*0.008*	*0.031*	*0.005*	**0.052**	0.409	0.743	0.637	CMV+ patients with in situ disease had higher cIL-10 than invasive (LN-, LN + , all); trended toward higher than benign
	CMV−	*0.019*	*0.011*	0.969	**0.135**	0.834	*0.010*	0.866	*0.012*	CMV− patients with Inv/LN− disease had higher cIL-10 than benign, in situ, or Inv/LN+
TNFα	All	0.468								
	CMV+	**0.196**	**0.196**	**0.073**	**0.073**	0.884	0.238	**0.090**	0.599	CMV+ patients with in situ and benign disease trended toward higher TNFα levels than invasive (LN-, LN + , all)
	CMV−	**0.055**	*0.011*	0.414	*0.047*	0.744	*0.040*	0.657	**0.091**	CMV− patients with invasive (LN-) disease had higher TNFα levels than in situ or benign and trended toward higher levels than invasive (LN +)
IL-6	All	0.409^a^								
	CMV+	**0.106**^a^	**0.107**	*0.024*	*0.025*	**0.111**	0.937	0.438	0.499	CMV+ patients with in situ disease had higher IL-6 than invasive (LN +) and trended toward higher than benign or invasive (LN-)
	CMV−	**0.115**^a^	*0.030*	0.262	**0.053**	0.501	0.175	0.696	0.316	CMV− patients with invasive (LN-) disease had higher IL-6 levels compared to in situ
cmvIL-10	CMV+	**0.108**	*0.027* (*0.014*^b^)	**0.117**	*0.027* (*0.020*^b^)	0.583	**0.082**	0.282	0.529	cmvIL-10 levels were higher in patients with in situ disease compared to invasive (LN-, all), even after adjustment for race

Italic text indicates a significant result (*p* < 0.05); bold text indicates a trend that could become significant with a larger sample size

^a^Kruskal-Wallis one-way ANOVA, ^b^Wilcoxon rank sum test

A similar trend was observed for TNFα (Fig. 2B). There were no significant differences in TNFα levels between disease groups for the overall study population (*p* = 0.468). When stratified for CMV status, however, clear differences emerged. In the CMV+ group, although the difference did not achieve statistical significance, TNFα levels were higher in patients with benign and in situ disease compared to Inv/LN− and Inv/LN+ (*p* = 0.196) (Fig. 2B). Patients with in situ disease had higher TNFα levels than Inv/LN+ or Inv/LN− and Inv/LN+ combined (*p* = 0.073) (Table 3). For CMV− patients, TNFα levels were highest in the Inv/LN− group compared to those with benign disease, in situ, or Inv/LN+ (*p* = 0.055) (Table 3).

For IL-6, no significant differences among disease groups for the overall study population were noted (*p* = 0.409) (Fig. 2C). Among CMV+ patients, the highest levels of IL-6 were in the in situ group (*p* = 0.106), and the difference between IL-6 levels in in situ and Inv/LN+ was significant (*p* = 0.024) (Table 3). For the CMV− group, we observed the highest levels of IL-6 in the Inv/LN− patients (*p* = 0.115) approaching statistical significance (Table 3). Among these CMV− patients, the Inv/LN− patients had significantly higher IL-6 levels than in situ group (*p* = 0.030) (Table 3).

**Fig. 3:**
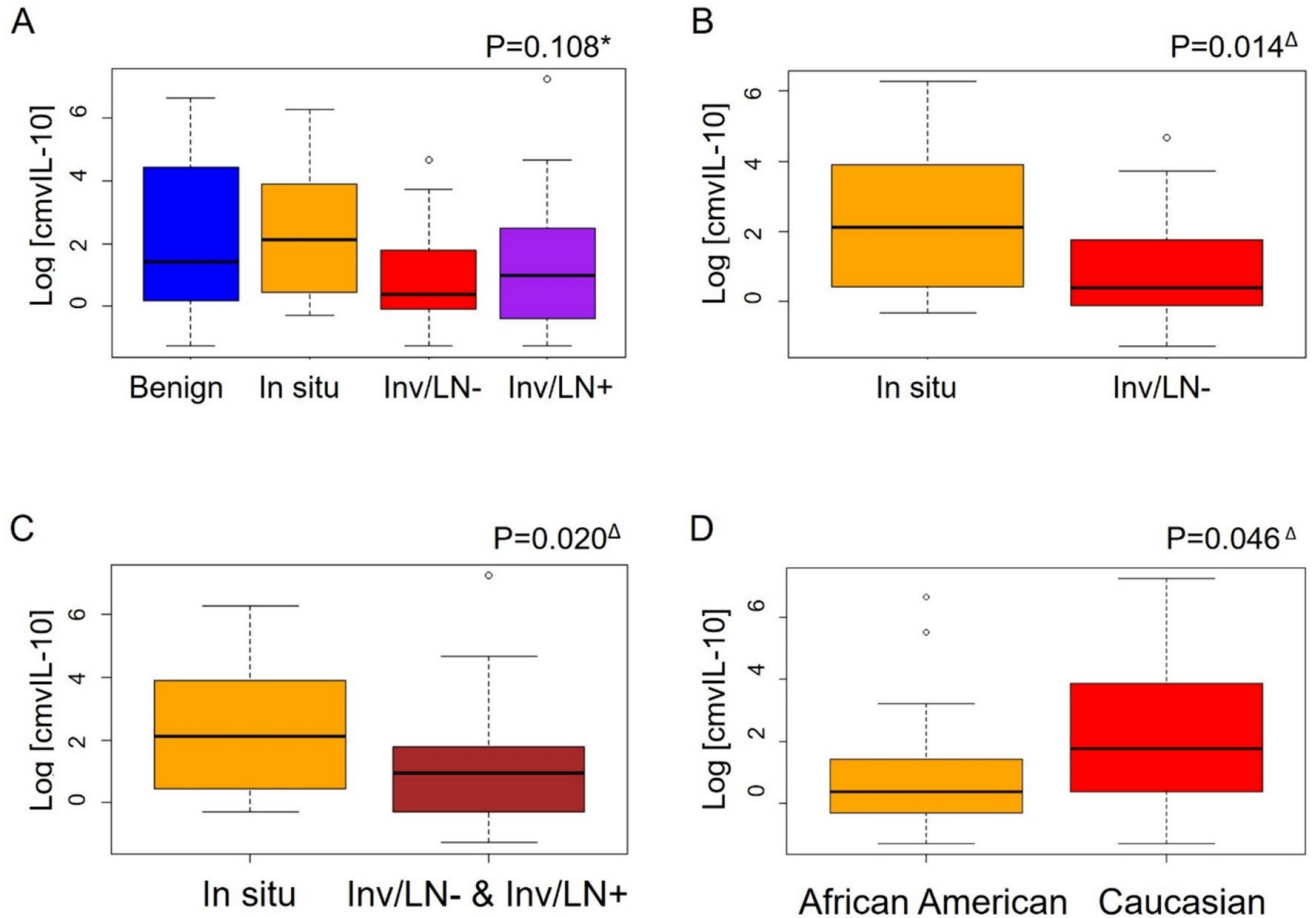
Log cmvIL-10 levels in CMV+ patients with breast disease. **A** Comparison of cmvIL-10 levels among all groups. **B** Comparison between in situ and invasive without lymph node involvement (Inv/LN−). **C** Comparison between the in situ group and the Inv/LN− and Inv/LN+ groups combined. **D** Comparison of cmvIL-10 levels between CMV+ patients in all disease groups by race (CMV+/African American, *n* = 20, CMV+/Caucasian, *n* = 52). Statistical analysis performed via *one-way ANOVA and ^Δ^Wilcoxon rank sum test

We next evaluated cmvIL-10 levels in CMV+ patients and found no significant difference in cmvIL-10 across all four disease groups (*p* = 0.108) (Fig. 3A). However, the in situ patients had significantly higher cmvIL-10 levels than the Inv/LN− (*p* = 0.014) (Fig. 3B) and the combined Inv/LN− and Inv/LN+ groups (*p* = 0.020) (Fig. 3C). The relative expression pattern for cmvIL-10 was reminiscent of that observed for cIL-10 levels in the CMV+ group (Fig. 2A). Notably, among CMV+ patients, the cmvIL-10 levels were significantly higher in Caucasian patients than in African American patients (*p* = 0.046) (Fig. 3D). There were no differences by race in either CMV+ or CMV− patients for the other cytokines evaluated (cIL-10, IL-6, or TNFα). Additionally, we evaluated whether sample collection time (at diagnosis vs after treatment) contributed to the differences in cytokine levels in either CMV+ or CMV− patients. We found there was no impact of sample collection time on differences in cytokine levels (Table 2).

**Fig. 4:**
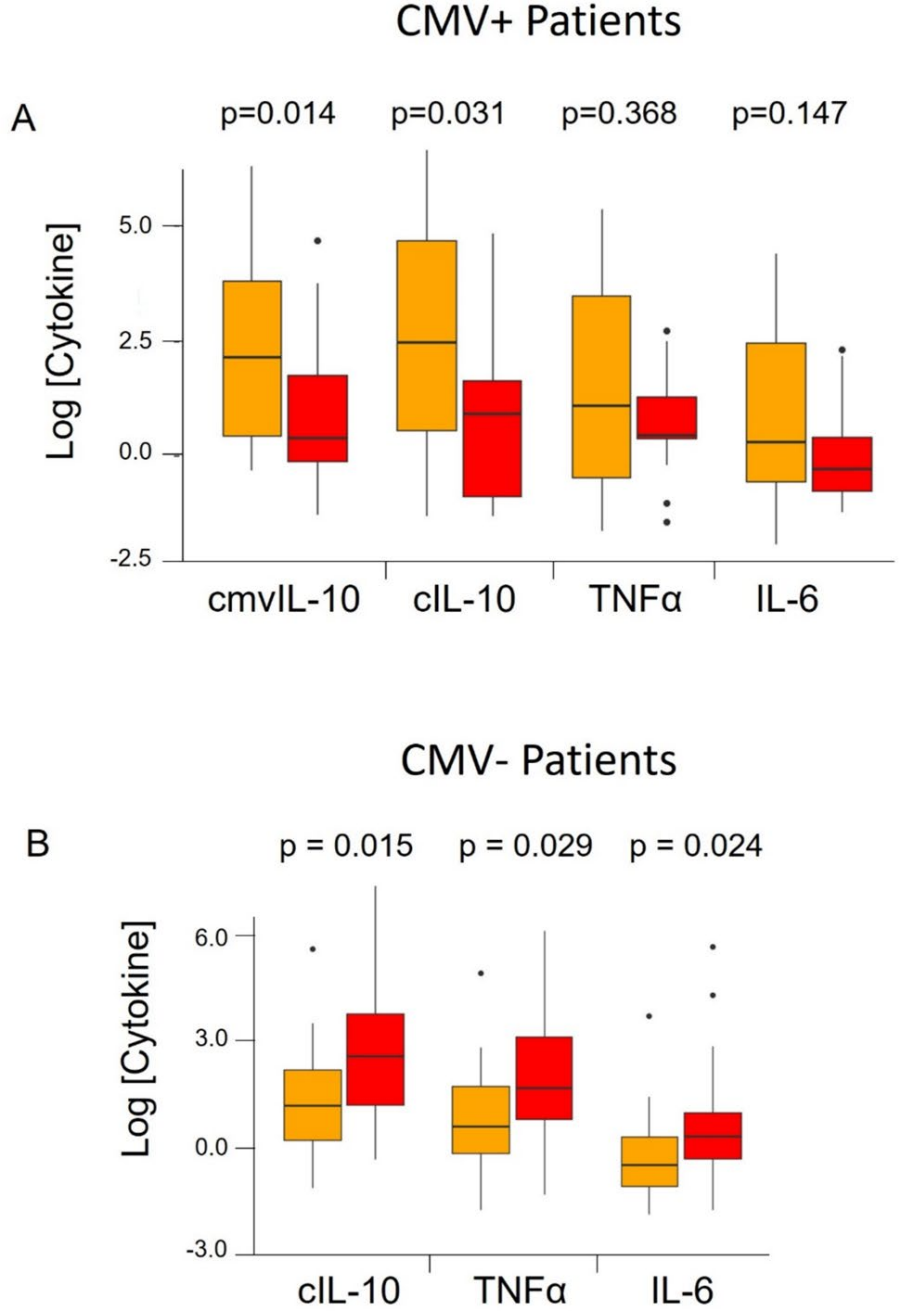
Log cytokine levels in CMV+ vs. CMV− patients with breast disease. **A** CMV+ patients, **B** CMV− patients. Orange, in situ; red, Inv/LN−. Statistical analysis by Wilcoxon rank sum test

The overall trend in this study was that cytokine profiles differed with respect to CMV serostatus (Fig. 4). In CMV+ patients, the highest cytokine levels were observed in the in situ group, with lower cytokine levels in the Inv/LN− group (Fig. 4A). CMV+ women with invasive carcinoma had the lowest levels of cmvIL-10 and cIL-10, which is the opposite of our initial prediction. In contrast, in CMV− women, the highest cytokine levels were in the Inv/LN− group, and levels in the in situ group were significantly lower (Fig. 4B). These results suggest that CMV infection impacts patterns of cytokine production in breast cancer.

## Discussion

We set out to investigate the relationship between CMV infection and breast disease diagnosis. We evaluated CMV IgG and cytokine levels in patients with different types of breast disease, and we hypothesized that cytokine levels would be higher in patients with more invasive disease. Our hypothesis was partially correct. Cytokine levels were highest in patients with invasive disease, but this was only true for individuals who were negative for CMV (Fig. 4B). A striking difference was observed in CMV+ patients, where cytokine levels were lowest in patients with more invasive disease (Fig. 4A). Our results demonstrate that the relationship between breast disease stage and cytokine levels is complex and dependent on CMV status. These findings suggest that CMV infection strongly influences patterns of cytokine production in breast cancer.

We did not observe any association between CMV IgG status and breast disease diagnosis. CMV serostatus has not been positively associated with breast cancer except in a few specific cases. One study of Iranian women found CMV IgG was more prevalent in women with breast cancer (*n* = 49) compared to healthy controls (*n* = 49, 94% vs 69% seropositivity, *p* = 0.002) [37]. A study of Iraqi women reported that 100% of women with malignant breast cancer (*n* = 60) were positive for CMV IgG, and 8.3% of those women were positive for CMV IgM, suggesting a recent infection or reactivation [38]. However, in the Iraqi study, CMV was also widespread in women with benign breast disease (*n* = 20, 95% CMV+) and in healthy controls (*n* = 10, 90% CMV+), indicating a high level of CMV infection in the Iraqi population overall. In contrast, a study of breast cancer survivors in the United Kingdom reported no significant differences in seroprevalence between survivors (*n* = 38, 44.4% CMV+) and healthy women (*n* = 27, 44.7% CMV+) [39]. Likewise, there was no difference in CMV seropositivity between Australian women with breast cancer (*n* = 208, 59% CMV+) and healthy controls (*n* = 168, 57% CMV+) [18]. The patient samples analyzed in our study were all collected in the US from women ages 25 to 69, and overall CMV seroprevalence was 50.3%, which is more comparable to the UK and Australian studies than the Iranian and Iraqi studies. CMV seroprevalence in the population varies widely based on factors such as age, race, ethnicity, geography, socioeconomic status, education level, and healthcare disparities [40].

While CMV seropositivity is not strongly associated with breast cancer, there are significant differences in CMV IgG levels between breast cancer patients and healthy women [18, 19, 39]. The same Australian study reported higher mean CMV IgG levels in women with breast cancer compared to controls, and this was notable because there was no difference in IgG levels specific for another common herpesvirus, Epstein-Barr virus (EBV) [18]. A long-term Norwegian study found that elevated CMV IgG levels preceded development of breast cancer, yet there was no correlation with EBV IgG levels [19]. The UK study reported higher CMV IgG levels in breast cancer survivors compared to healthy controls, although the difference was not statistically significant [39]. Similarly, we saw a trend toward higher IgG levels in women with invasive carcinoma (Inv/LN−) compared to women with benign breast disease or in situ carcinoma (Fig. 1), but our sample size for each diagnosis was relatively small. Of note, we observed that CMV IgG levels were lowest in patients with the most invasive disease (Inv/LN+), suggesting that the relationship between CMV IgG levels and disease severity is not a linear correlation. Tumor progression is influenced by many factors, and our results suggest that these factors may include the presence of CMV, the status of the infection, and the level of immune response to the virus.

One focus of this study was to examine cmvIL-10 levels in breast cancer to evaluate any potential use as a biomarker for tumor progression or disease severity. Although we expected higher cmvIL-10 levels to correlate with immune suppression and therefore, more severe or invasive disease diagnosis, we found just the opposite. We observed the highest cmvIL-10 levels in individuals with in situ tumors (Fig. 3A). The level of cmvIL-10 was lower in patients with invasive tumors (Fig. 3B, C), which could indicate a change in CMV activation or replication when tumor cells break out of the encapsulated environment to invade the surrounding tissue. One possibility is that high cmvIL-10 levels in patients with an in situ diagnosis reflect a period of active virus replication, perhaps due to immune suppression or other stressors, creating a perfect storm of conditions that allow tumor cells to break through the ductal walls and invade surrounding breast tissue. Once this breakthrough event happens, an inflammatory immune response may be induced, and the tumor microenvironment would likely change dramatically. As the immune system rebounds and fights off virus infection, the immune cells may be unable to reverse the tumor invasion that has already occurred. This surge of immune activity could account for the lower levels of both cmvIL-10 and human cIL-10 observed in patients with invasive tumors. In glioblastoma multiforme (GBM) tumors, glioma cancer stem cells were found to be CMV+ and secrete cmvIL-10, influencing monocyte function, creating an immune suppressive environment, and promoting tumor progression [32].

Another possible explanation for our results is that lower levels of cmvIL-10 in invasive disease are due to the activity of neutralizing antibodies that eliminate cmvIL-10 from the bloodstream. Neutralizing antibodies to cmvIL-10 have been detected in both humans [36] and Rhesus macaques [41, 42], but any connection between cmvIL-10 neutralizing antibodies and tumor progression remains to be explored. There are many factors that contribute to tumor progression, and not all lesions do progress from in situ to invasive. Some in situ lesions remain localized for years without ever becoming invasive, and there is currently no reliable test to identify lesions with invasive potential [43]. Progress is being made toward identifying gene signatures that predict likelihood of invasion [44, 45], but more research is needed to understand the complex processes that govern tissue invasion.

We also found that cmvIL-10 levels were significantly lower among African American study participants compared to Caucasian participants (Fig. 4D). This finding is consistent with previous reports that CMV seroprevalence is higher in non-Caucasian populations [46, 47]; however, it is unclear whether the differences observed here may relate to differences in tumor status, CMV replication, immune function, or other factors. Notably, there was no difference in human cIL-10, TNFα, or IL-6 levels between African American and Caucasian study participants. A study of healthy adults in California reported higher levels of C-reactive protein (CRP) and IL-6 in African Americans compared to Caucasians, but no difference in cIL-10 or TNFα levels [48]. In this study, we also did not observe any correlation between cmvIL-10 levels and age, although a correlation with age was reported in a Taiwanese study of CMV+ patients with *Aspergillus* infection [49].

The patient plasma samples analyzed here represent single snapshots in time from individual patients with the same diagnosis. There were no serial samples from the same patient as disease progressed, so we did not have the opportunity to evaluate changes over time. However, even with our small sample size, we did see highly significant differences in cytokine levels between diagnosis groups with opposite profiles in CMV+ vs. CMV− patients. These results strongly suggest that either CMV has some influence in the progression of breast cancer, or conversely, that the progression of cancer has some influence over CMV reactivation and latency.

The majority of plasma samples in this study were drawn at the time of diagnosis. Among patients with invasive carcinoma with (Inv/LN+) or without lymph node involvement (Inv/LN−), some samples were collected after treatment with chemotherapeutic agents. Chemotherapy can suppress the immune system, making patients more susceptible to infections, including CMV reactivation. However, there is currently no evidence to suggest that chemotherapy directly triggers CMV reactivation. One report found no evidence of CMV reactivation following chemotherapy in a study of 93 patients with solid organ tumors in Turkey [50]. Our analysis found no significant impact of treatment on cytokine levels (Table 2), further supporting the notion that differences in cytokine levels are primarily due to CMV status and disease diagnosis.

It is also worth noting that this study relied on plasma from women with a diagnosis of breast disease, not a tissue biopsy sample from the site of the lesion. Direct analysis of tumor tissue, typically by immunohistochemistry staining or molecular techniques, can provide valuable information about gene expression patterns and protein localization and abundance, identifying potential disease markers or therapeutic targets within the tumor microenvironment. A logical future extension of this study would be to further stratify diagnoses by hormone receptor expression or other tumor markers. Regrettably, we did not have this information for all patient samples analyzed here. On the other hand, biopsy samples are typically processed by formalin fixation, paraffin embedded tissue, which may result in masking or modification of some antigens, formation of artifacts, and degradation of nucleic acids. By comparison, plasma samples reflect the state of the whole body, and it is remarkable to see such profound differences in systemic cytokine patterns between CMV+ and CMV− women with different diagnoses of breast disease. A particular strength of this study is that we stratified the data by diagnosis, making a distinction between invasive carcinoma with lymph node involvement (Inv/LN+) or without (Inv/LN−). Our analysis revealed stark differences between CMV+ and CMV− cytokine profiles not only between in situ and invasive disease, but between invasive with or without lymph node involvement (Fig. 2A, Table 3). Further analysis of CMV status and plasma cytokines may result in profiles that could be combined with other biomarkers such as circulating tumor DNA in liquid biopsies [51] to actively monitor tumor dynamics and response to treatment.

CMV establishes lifelong latent infection in the host. It is now widely understood that the viruses, bacteria, and fungi that make up the human microbiome play a significant role in human health and disease, and they can profoundly influence tumor development [52, 53]. In a murine breast cancer model, the microbiome profoundly impacted tumor development [54]. Tissue-resident intracellular bacteria enhanced survival of circulating tumor cells and strongly promoted metastatic spreading to the lungs [54]. The number of lung metastases were reduced when mice were administered antibiotics, confirming the role of the tumor microbiota in metastasis formation. In addition, commensal fungi have been observed to colonize pancreatic tumors, stimulating production of IL-33 and suppressing anti-tumor immune responses [55]. Our results clearly indicate a relationship between CMV status and systemic cytokine response in women with breast cancer, but these observations lead to more questions than they answer. In women with CMV, cytokine levels were higher in in situ and lower in invasive disease (LN−/LN+). But in women without CMV, cytokine levels were lower in situ and higher in invasive disease (LN−/LN+). We do not yet know whether this indicates that CMV infection may suppress anti-tumor responses, or if patients with CMV already have elevated cytokine levels due to virus infection, and those levels decrease in concert with tumor progression. While a profound difference in cytokine production was observed here between CMV+ and CMV− patients, more research is needed to fully understand the impact of CMV on breast cancer.

## Data Availability

No datasets were generated or analysed during the current study.
